# Association Equilibria of Organo-Phosphoric Acids with Imines from a Combined Dielectric and Nuclear Magnetic Resonance Spectroscopy Approach

**DOI:** 10.1021/acs.analchem.0c04669

**Published:** 2021-02-18

**Authors:** Christian Dreier, Leon Prädel, Amelie A. Ehrhard, Manfred Wagner, Johannes Hunger

**Affiliations:** † Max Planck Institute for Polymer Research, Department for Molecular Spectroscopy, Ackermannweg 10, 55128 Mainz, Germany

## Abstract

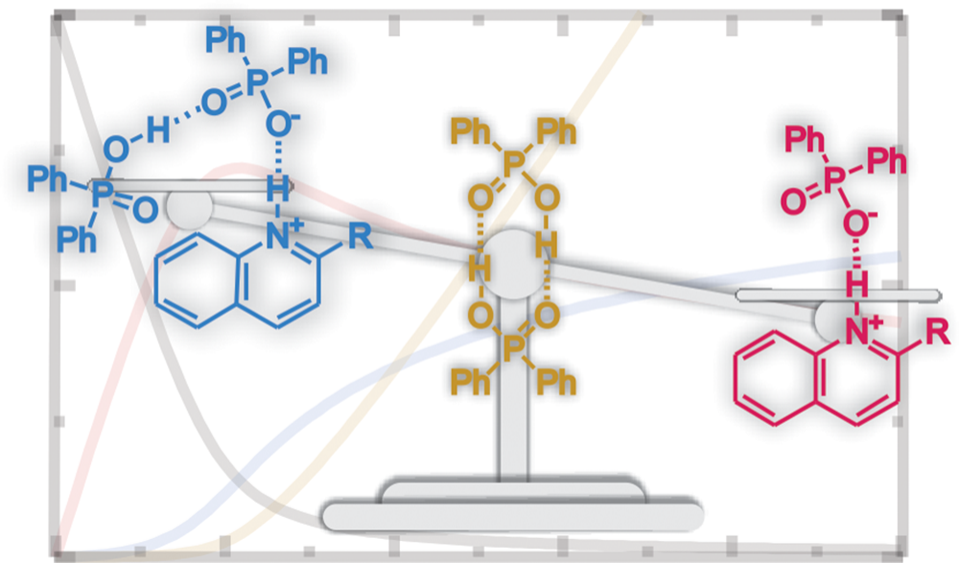

Aggregates formed between organo-phosphoric acids and imine bases in aprotic solvents are the reactive intermediates in Brønsted acid organo-catalysis. Due to the strong hydrogen-bonding interaction of the acids in solution, multiple homo- and heteroaggregates are formed with profound effects on catalytic activity. Yet, due to the similar binding motifs—hydrogen-bonds—it is challenging to experimentally quantify the abundance of these aggregates in solution. Here we demonstrate that a combination of nuclear magnetic resonance (NMR) and dielectric relaxation spectroscopy (DRS) allows for accurate speciation of these aggregates in solution. We show that only by using the observables of both experiments heteroaggregates can be discriminated with simultaneously taking homoaggregation into account. Comparison of the association of diphenyl phosphoric acid and quinaldine or phenylquinaline in chloroform, dichloromethane, or tetrahydrofuran suggests that the basicity of the base largely determines the association of one acid and one base molecule to form an ion-pair. We find the ion-pair formation constants to be highest in chloroform, slightly lower in dichloromethane and lowest in tetrahydrofuran, which indicates that the hydrogen-bonding ability of the solvent also alters ion-pairing equilibria. We find evidence for the formation of multimers, consisting of one imine base and multiple diphenyl phosphoric acid molecules for both bases in all three solvents. This subsequent association of an acid to an ion-pair is however little affected by the nature of the base or the solvent. As such our findings provide routes to enhance the overall fraction of these multimers in solution, which have been reported to open new catalytic pathways.

## Introduction

The hydrogen-bond formed between Brønsted acids and Brønsted bases is crucial for the catalytic activation in Brønsted acid organo catalysis.^1−4^ In organic aprotic solvents, the common reaction medium for such catalyses, the hydrogen-bond between the acid and base is characterized by the acidic proton residing in a shallow potential minimum between the two molecules^5,6^ and can be thus classified as strong hydrogen-bond.^7^ In catalysis, the bonding strength and the hydrogen-bonding potential critically influence the catalytic activity:^6^ For instance, organo-catalytic reaction rates have been demonstrated to correlate with the acidity of the catalyst, while enantioselectivities in asymmetric catalysis have been suggested to hardly scale with acidity.^8^ However, not only does the acidity of the catalyst critically affect the interaction but also the hydrogen-bond is susceptible to interactions and fluctuations of the solvent.^9^ As a consequence, catalytic efficiencies have been reported to markedly vary with the solvent:^10−13^ For the organo-phosphoric acid-catalyzed transfer hydrogenation, the solvents chloroform (CHCl_3_) and dichloromethane (DCM) have been shown to provide high enantioselectivities and yields.^10,12,13^ Despite good stereocontrol, yields have been reported to be lower when tetrahydrofuran (THF) is used as a solvent.^10,14,15^ In highly polar acetonitrile the enantioselectivity is drastically reduced.^14,16^ Despite progress in resolving the reaction mechanism based on experiments^17,18^ and theory,^19−21^ understanding solvent effects has remained challenging.^22^

**Scheme 1:**
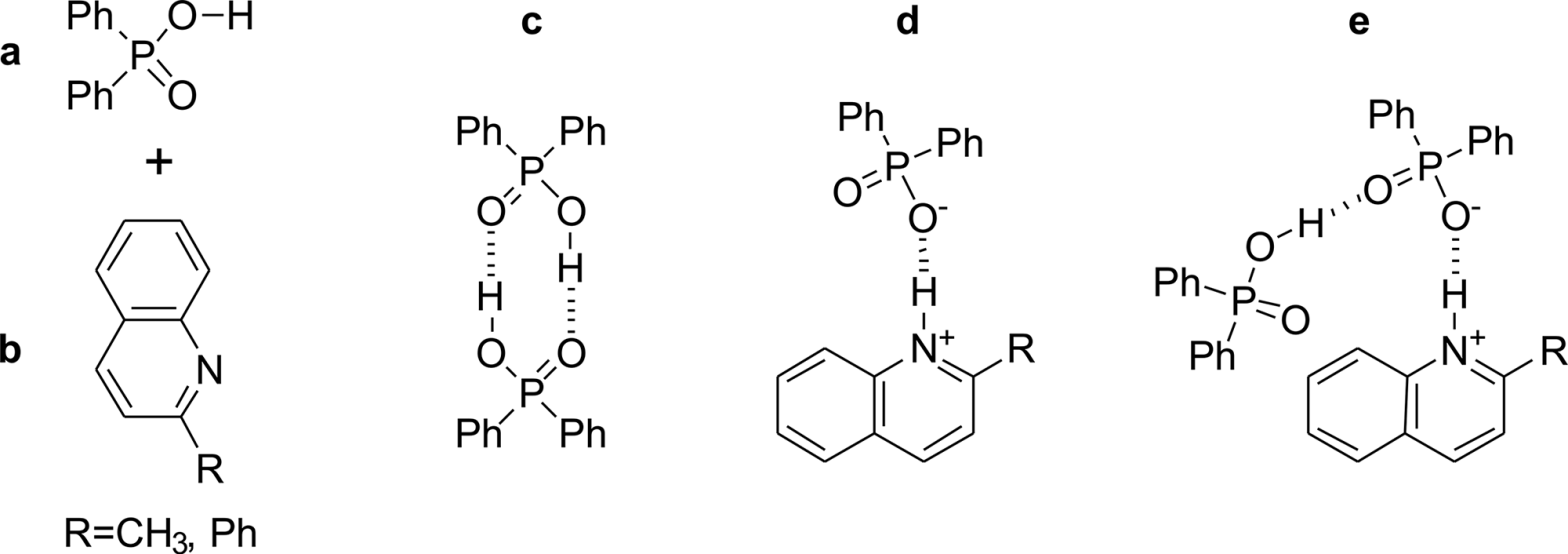
In Solutions of (a) Diphenylphosphoric Acid and (b) Chinoline Bases (R = CH_3_: Quinaldine, R = Ph, Phenylquinaldine) Different Aggregates Form, Including (c) Acid Homodimers, (d) Ion-Pairs, and Multimers As one example for various conceivable multimeric structures, a diphenylphosphoric acid donating a hydrogen-bond to an ion-pair within a trimer is displayed in (e).

The challenge in understanding such solvent effects is further exacerbated by the complexity of the solutions relevant to catalysis, which consist of several components: solvent, reactants, and catalysts. The structures of the acid catalyst and the bases relevant to the present work are shown in Scheme 1a and b, respectively. The molecular-level interaction between the different components and the subtle balance between the different homo- and heteroaggregation strengths of all components leads to the formation of various aggregates in solution at catalytically relevant temperatures.^5,23−26^ Brønsted acids in aprotic solvents tend to aggregate in solution (homodimers, Scheme 1c).^25−27^ In the presence of bases, we could recently show that not only heterodimers consisting of an acid, which binds and transfers a proton to the base (ion-pairs, IPs, Scheme 1d), form in solution, but also multimers (Ms, Scheme 1e) composed of the imine and more than one phosphoric acid molecule are present.^5,23^ Given that such acid base aggregates are the reactive intermediates in catalysis, they critically influence catalytic pathways, which has been demonstrated both computationally^28,29^ and experimentally.^30^ In fact, only recently was it shown that multimeric aggregates can be used for new catalytic pathways.^31^ Such multimeric aggregates have further been shown to affect reaction rates and can even reverse the enantioselectivity.^32^ As such, knowledge of the nature of the aggregates and their abundance can help in understanding the catalytically active species and thereby optimize the catalytic conditions.^33,34^ Yet, rapid fluctuations of these aggregates and the similarity of their bonding motifs makes it challenging to quantify the different aggregates in solution.

Here, we show that using the nuclear magnetic resonance (NMR) chemical shift of the protons of the base together with rotational relaxation modes of the dipolar aggregates as detected with dielectric relaxation spectroscopy (DRS) allows for a discrimination of the different aggregates. In analogy to our previous study, we use diphenyl phosphoric acid (DPP) as a model for organo-phosphoric acid catalysts.^5,14,23,35^ We study the interaction of DPP with two different bases, which are commonly used in phosphoric acid-catalyzed transfer hydrogenations:^1^ quinaldine (Qu) and 2-phenylquinoline (PhQu). To discern the role of the solvent, we study solutions of these molecules in CHCl_3_, DCM, and THF. To disentangle different aggregates formed in solution, we advance our previous approach using a combination of NMR and DRS spectroscopy:^23^ We determine the association equilibria from a simultaneous analysis of the NMR chemical shifts of the imine base, which sensitively reports on the motionally averaged electronic density of all bases in solution, and the dielectric relaxation strength of acid–base aggregates, which allows for quantitative assessment of the acid–base dimers and multimers in solution via the rotational relaxation of these dipolar aggregates. We demonstrate that only the joint experimental information can disentangle all relevant equilibria. Our results show that the basicity of the imine predominantly affects bimolecular acid–base aggregation. The observed effect of the solvent cannot be explained by the solvent’s dielectric constant, rather the solvent’s hydrogen-bonding ability appears to be decisive. Conversely, multimer formation depends only weakly on the base and the solvent.

## Materials and Methods

2-Phenylquinoline (PhQu, Alfa Aesar 99%) and diphenyl phosphoric acid (diphenyl phosphate, DPP, Sigma-Aldrich, 99%) were used as received. Quinaldine (Qu, Sigma-Aldrich, 95%) was dried over 4 Å molecular sieves and filtered using a 0.2 μm Omnipore membrane filter prior to use. The solvents chloroform (CHCl_3_, Fisher Scientific, HPLC grade), dichloromethane (DCM, Fisher Scientific, HPLC grade), tetrahydrofuran (THF, Fisher Scientific, HPLC grade), deuterated chloroform (CDCl_3_, Sigma-Aldrich, 99.8%), deuterated DCM (CD_2_Cl_2_, Deutero, 99.6%), and deuterated THF (C_4_D_8_O, Carl-Roth, 99.5%) were either taken from fresh bottles or dried over 4 Å molecular sieves and filtered using a 0.2 μm Omnipore membrane filter.

Stock solutions of imine (0.2 mol L^–1^) and DPP (1.0 mol L^–1^) were prepared gravimetrically using volumetric flasks. Samples were prepared by mixing the appropriate volumes of stock solutions of the imine and DPP with pure solvent using graduated glass pipettes, assuming ideal mixing volumes. All investigated solutions have a constant imine concentration of 0.1 mol L^–1^, while the concentration of DPP, *c*_DPP_, varied from 0.01 to 0.5 mol L^–1^. These concentrations were chosen such that sufficiently high relaxation amplitudes can be obtained in the DRS experiments. For the NMR experiments 1 mL of each sample was prepared using the deuterated solvents. For DRS experiments only nondeuterated solvents were used to prepare 2.5–4.5 mL total sample volume.

NMR spectra were measured using a 300 MHz AVANCE III Bruker spectrometer (Bruker TOPSPIN 2.1 software version). ^1^H- and COSY-spectra were recorded and used for peak assignment (^1^H: 16 scans, 13.3 μs long π/2-pulse, spectral width 6172 Hz; COSY: 1 scan 13.3 μs long π/2-pulse). All NMR experiments were performed at 298.15 ± 0.5 K. All spectra were referenced to the residual solvent peak (C*H*Cl_3_^1^H: 7.26 ppm,^36^ C*H*DCl_2_^1^H: 5.32 ppm,^37^ THF-*d*_7_^1^H: 3.58 ppm^37^). The insensitivity of the chemical shift of some protons of the studied bases to an excess of base suggests that the shifts of the solvents residual peaks are hardly affected by DPP (see Figure S1, Supporting Information, SI). The spectra were analyzed with the multiplet analysis tool of MestReNova (Version 14.0.1).

DRS^38,39^ measures the rotational relaxation of dipolar aggregates in solution, by recording the polarization of the sample in an external oscillating electric field. This polarization can be expressed as the frequency (ν) dependent complex permittivity ε̂(ν), with ε^′^(ν) the real part and ε^′′^(ν) the imaginary part of the complex permittivity.
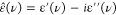
1All complex permittivity spectra were recorded using an Anritsu MS4647A Vector Network Analyzer at frequencies ranging from 10 MHz to 125 GHz at ambient temperature (295 ± 1 K). To cover this broad frequency range, a combination of three experimental reflectometer geometries was used. A cutoff type coaxial cell^40,41^ was used at frequencies from ∼10 MHz to ∼2 GHz. At ∼1 GHz to ∼50 GHz an open-ended, 1.85 mm connector based, coaxial cell was used.^42,43^ Frequencies from 56 to 125 GHz were covered with a coaxial reflectometer based on the Anritsu 3744A mmW external frequency converter module.^44,45^ Note that the exact frequency ranges covered by each reflectometer vary, as the scatter of the data depends on the sample properties. The reflectometers were calibrated using air, ethanol,^46^ and conductive silver paint (or 22.65 wt % NaCl aqueous solution^47^ for the cutoff probe).^14^

## Results and Discussion

### Methodology to Determine Association Equilibria

**Figure 1:**
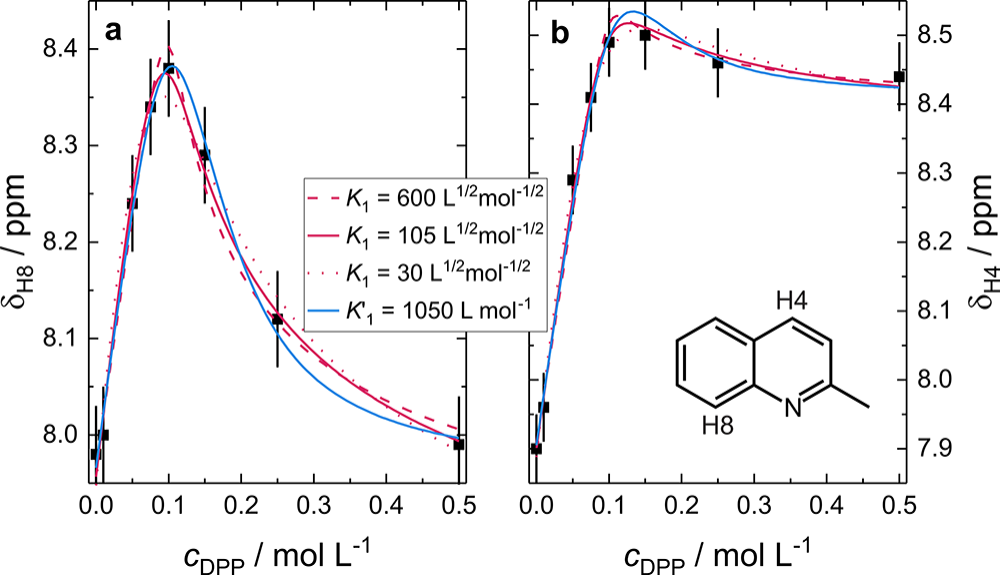
Chemical shift of (a) H8 and (b) H4 for a solution of 0.1 mol L^–1^ Qu in CDCl_3_ as a function of c_DPP_. Symbols show experimental data and error bars were estimated to ±0.05 ppm to account for systematic errors due to medium effects (see text). The molecular structure of Qu together with the proton labels are shown as an inset in panel (b). Magenta lines show fits using eq 2 to the data with the equilibria as defined in eqs 3 and 4. For the red dotted and dashed lines the value of *K*_1_ was constrained to 30 and 600 L^1/2^ mol^–1/2^, respectively. Solid blue lines show the fit according to eq 2, without taking DPP dimerization into account.^23^

To determine the interaction of DPP with organic bases, NMR spectroscopy is a powerful tool to interrogate the electronic environment of the base. In general, as protonation of the aromatic base alters the electron density distribution of the base, a downfield field shift of the base’s aromatic protons is indicative of proton transfer to the base. As such, NMR titration can be used to infer protonation equilibria^48,49^ (and association strengths).^23,50,51^ In Figure 1 we show the variation of the chemical shift (δ) for two selected protons of Qu (*c*_Qu_ = 0.1 mol L^–1^) in CDCl_3_ as a function of *c*_DPP_. At *c*_DPP_ < 0.1 mol L^–1^ the values of δ increase monotonically with increasing *c*_DPP_, providing evidence for protonation of Qu by DPP (and formation of QuH^+^-DPP^–^ ion-pairs, IPs^14,23^). At *c*_DPP_ > 0.1 mol L^–1^ the chemical shifts decrease with increasing *c*_DPP_, which provides evidence for the formation of a different molecular aggregate at high DPP concentrations. We have assigned these aggregates to multimers (Ms) consisting of one Qu and more than one DPP molecule, in which an additional acid molecule donates a hydrogen-bond to an already existing Qu-DPP complex.^23^ This decrease of δ is most pronounced for protons in the vicinity of the N atom of Qu, which we have ascribed to the association of the additional DPP molecules in the vicinity of the protonated N atom of Qu.^23^

To obtain the composition dependent equilibrium concentrations of aggregates, here [*j*] (*j* = Qu, DPP, IP, or M), the experimentally determined chemical shifts have to be modeled: The observed motionally averaged chemical shift, δ, of Qu’s protons can be expressed as the concentration weighted average of the chemical shift of the underlying aggregates, *δ*_*j*_:
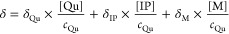
2To determine the equilibrium concentrations, association equilibria have to be assumed. In our earlier study^23^ we used for the sake of simplicity, the formation of ion-pairs from DPP and Qu (Qu + DPP ⇆ IP) and approximated the multimers as trimers (IP + DPP ⇆ M) with the apparent association constants *K*_1_^′^ = [IP]/([Qu][DPP]) and *K*_2_^’^=[M]/([IP][DPP]). These two equilibria sufficed to described the data. Yet these equilibria did not take the association of DPP^25−27^ into account, while in aprotic solvents Brønsted acids are nearly exclusively present in aggregated form.^26^ To take the association of DPP into account, we assume in the present work formation of DPP dimers (2 DPP ⇆ DPP_2_), which dissociate prior to the aggregation with Qu or IP, and compare the findings to our earlier approach:
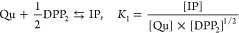
3
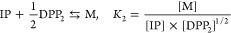
4To this end, we simultaneously fit eq 2 to the chemical shifts of the protons H4 and H8—the aromatic protons, which are most sensitive to multimer formation. Together with mass conservation, eqs 2–4 describe the experimentally determined values of δ very well (see magenta solid lines in Figure 1), using the association equilibria *K*_1_ and *K*_2_ and the chemical shifts of Qu δ_Qu_, the IP δ_IP_, and M *δ*_M_ for both protons (H4 and H8) as adjustable parameters (for parameters see Table 1). However, also neglecting DPP dimerization describes the data nearly equally well and for the presently studied samples (Qu and PhQu in THF, DCM, and CHCl_3_) the sum of the squared deviations of the fits does not provide evidence for eqs 3 and 4 or the equilibria of ref (23) to better describe the experimental data (see also SI Figure S2). The insensitivity of the fit to the different association models can be explained by the fit parameters being correlated: For instance, the experimental data can be almost equally well-modeled with an equilibrium constant *K*_1_ ranging from 30 to 600 L^–1/2^ mol^–1/2^ (dashed and dotted lines in Figure 1) as an increased association strength can be compensated in the fit by a decreased value of δ_IP_ (the fitting parameters *K*_1_ and δ_IP_ are anticorrelated, see SI Figure S3). As the experimentally measured variation of δ is not solely due to molecular association, but also contains a variation due to medium effects^52^ due to the progressive substitution of the pure solvent by the added acid, the experimental data are prone to systematic errors, in particular at high concentrations of acid. Thus, we estimate the overall uncertainty in the chemical shifts to ±0.05 ppm. Together, these uncertainties prevent an exact determination of the chemical equilibria for the presently studied samples based on only the NMR chemical shifts.

**Table 1:** Chemical Shifts of Free Quinaldine or 2-Phenylquinoline, δ_Qu_, Ion-Pairs, δ_IP_, and Multimers, δ_M_, for Protons H4 and H8 Together with the Equilibrium Constants *K*_1_ and *K*_2_ As Obtained from Fitting Eqs 2–4 to the NMR and DRS Data^a^

	δ_Qu_ (ppm)	δ_IP_ (ppm)	δ_M_ (ppm)	*K*_1_ (L^1/2^ mol^–1/2^)	*K*_2_ (L^1/2^ mol^–1/2^)
Qu					
CDCl_3_	H4: 7.90 ± 0.02	H4: 8.68 ± 0.02	H4: 8.24 ± 0.04	79 ± 18	3.1 ± 0.2
	H8: 7.96 ± 0.02	H8: 8.61 ± 0.02	H8: 7.51 ± 0.04		
CD_2_Cl_2_	H4: 8.05 ± 0.02	H4: 8.66 ± 0.03	H4: 8.47 ± 0.03	36 ± 6	6.1 ± 0.4
	H8: 7.95 ± 0.02	H8: 8.63 ± 0.03	H8: 7.81 ± 0.03		
THF	H4: 8.02 ± 0.03	H4: 9.07 ± 0.04	H4: 8.50 ± 0.06	8.2 ± 0.9	3.5 ± 03
	H8: 7.94 ± 0.03	H8: 8.77 ± 0.04	H8: 7.65 ± 0.06		
PhQu					
CDCl_3_	H4: 8.17 ± 0.03	H4: 8.77 ± 0.06	H4: 8.75 ± 0.05	13 ± 3	6.7 ± 0.6
	H8: 8.25 ± 0.03	H8: 8.95 ± 0.06	H8: 8.24 ± 0.05		
CD_2_Cl_2_	H4: 8.24 ± 0.03	H4: 8.76 ± 0.04	H4: 8.82 ± 0.07	11 ± 2	2.6 ± 0.3
	H8: 8.13 ± 0.03	H8: 8.91 ± 0.04	H8: 8.05 ± 0.08		
THF	H4: 8.18 ± 0.03	H4: 8.55 ± 0.06	H4: 9.29 ± 0.06	6.2 ± 3.8	3.3 ± 0.7
	H8: 8.13 ± 0.03	H8: 8.22 ± 0.06	H8: 8.82 ± 0.06		

a Errors correspond to a 10% increase in the sum of the squared deviations (see also SI eq S4).

To lift the ambiguity in modeling the NMR chemical shifts, we use a second method that allows us to quantitatively determine the equilibrium concentrations of the different species. We use DRS, which is sensitive to the rotational relaxation of molecular dipoles.^53^ For the samples of the present study the solvent, IPs, and Ms are the predominant dipolar species that contribute to the dielectric spectra. (Note that DRS peak amplitudes scale with concentration and the squared electrical dipole moment, see SI eq S3. As such, the contribution of the rotational relaxation of weakly dipolar DPP and Qu to the spectra is negligible.)^14^ The contribution of the three predominant dipolar species to the spectra can be disentangled via their relaxation time (i.e., peak position) in the DRS spectra, as the relaxation time scales with viscosity and hydrodynamic volume: The larger the volume of the rotating dipolar species the longer its relaxation time, i.e., the lower its relaxation frequency.^54^ For uncorrelated molecular motion, each relaxation gives rise to a dispersion in the frequency dependent permittivity ε′(ν) and a peak in the dielectric loss spectrum ε′′(ν).

**Figure 2:**
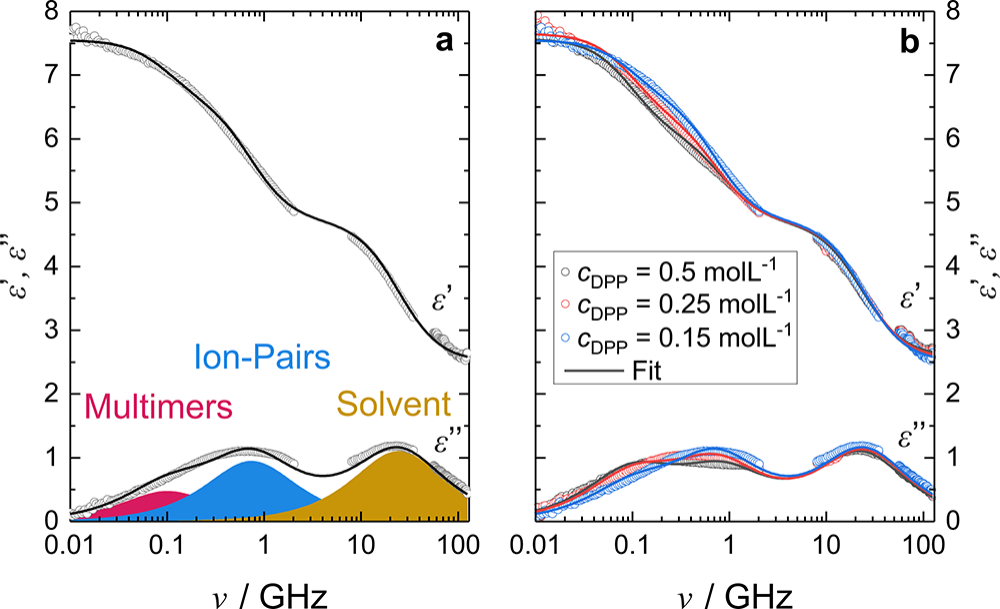
(a) Dielectric permittivity, ε′, and dielectric loss, ε′′, spectra for a solution of *c*_Qu_ = 0.1 mol L^–1^ and *c*_DPP_ = 0.15 mol L^–1^ in CHCl_3_. Symbols show experimental data and solid lines show the fit using three Debye-type relaxations (SI eq S2). The contribution of the three Debye relaxations to the dielectric loss are shown as shaded areas (magenta: multimers, blue: ion-pairs, dark-yellow: solvent). (b) Experimental spectra (symbols) together with the fits (solid lines) for solutions of 0.1 mol L^–1^ Qu in CHCl_3_ with different concentrations of DPP.

In Figure 2a we show the dielectric permittivity spectrum for a solution of Qu (0.1 mol L^–1^) and DPP (0.15 mol L^–1^) in CHCl_3_. The permittivity spectrum shows a pronounced dispersion within the studied frequency range, and the dielectric loss spectrum exhibits a somewhat narrow peak at high frequencies and an asymmetric broad peak at lower frequencies. The asymmetry of the lower frequency peak is indicative of multiple relaxations contributing to this peak. A combination of three Debye-type relaxations describes the experimental spectra very well (black solid line in Figure 2a),^23^ and we therefore use this model to decompose the spectra into three relaxations (see SI eq S2). The contributions of the three individual relaxations to ε′′(ν) are shown as magenta, blue, and dark-yellow shaded areas in Figure 2a. The high-frequency relaxation centered at ∼20 GHz can be assigned to the solvent.^55^ Similar to our previous work, we assign the two relaxations at lower frequencies to ion-pairs (centered at ∼700 MHz) and multimers (∼100 MHz), which both give rise to a broad spectral feature at low frequencies.^23^ Upon increasing DPP concentration the solvent relaxation at ∼20 GHz is rather unaffected (Figure 2b). Conversely, the dielectric loss at ∼700 MHz decreases, while the loss at ∼100 MHz increases. This shift of the loss peak to lower frequencies is indicative of the formation of multimers at the cost of ion-pairs.

The relaxation amplitudes, *S*_*j*_, as extracted from the relaxation model are directly related to concentration [*j*] and squared electrical dipole moment μ_*j*_^2^ of the species in solution (see eq S3, SI).^14,23,56^ Thus, in order to quantify the equilibrium concentrations of all species, their electrical dipole moment *μ*_*j*_ is required. Here, we extract the value of μ_M_ and μ_IP_ from the relaxation amplitudes *S*_M_ and *S*_IP_ for the sample with the highest concentration of DPP. This is achieved by assuming that all Qu in solution form either IPs or Ms, which can be justified given the 5-fold excess of acid. Further, we assume μ_M_ = μ_IP_, which is supported by our previous experiments and *ab-inito* calculations.^14,23^ Thus, we extract the equilibrium concentrations [*IP*] and [*M*] from the dielectric relaxation strengths (see SI for details). These concentrations are used to constrain the fit of the NMR chemical shifts with eq 2. Since the accuracy of the determined relaxation strengths (and hence the concentrations) is typically a few percent (∼2%)^57^ of the static dielectric constant, the derived equilibrium concentrations are most accurate at elevated concentrations where a sufficiently high concentration of dipolar aggregates (IP or M) is formed. To this end, we use the DRS determined IP and M concentrations at three DPP concentrations with an excess of DPP. The concentration of DPP is chosen such to aim at [IP] > [M], [IP] ≈ [M], and [IP] < [M] for the three studied concentrations.

**Figure 3:**
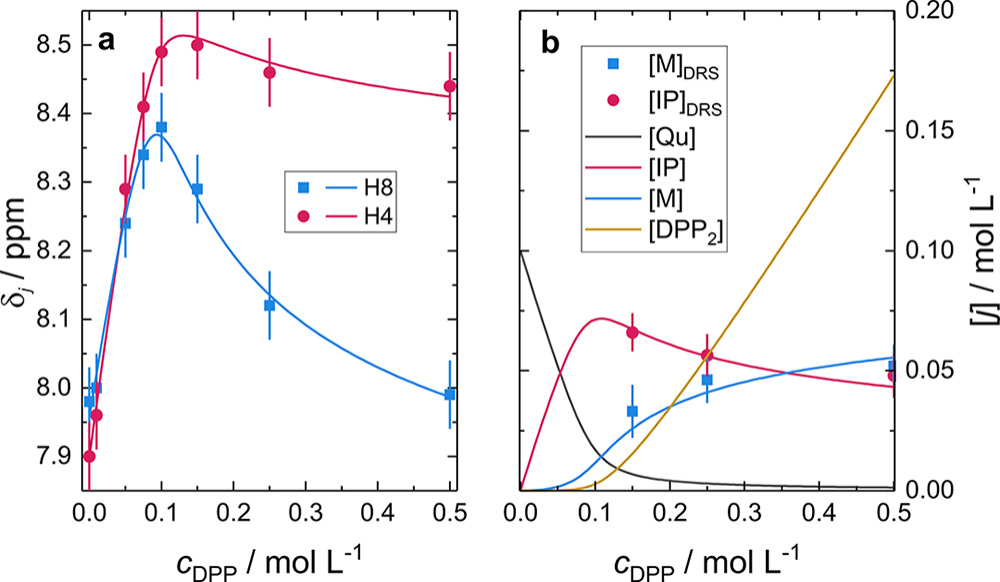
Combined fit of the NMR and DRS results for solutions of 0.1 mol L^–1^ Qu in chloroform. (a) NMR chemical shift (symbols) of H4 and H8 and (b) equilibrium concentrations of multimers and ion-pairs (symbols) as extracted from the DRS experiments. Solid lines in panel (a) show the fit of the NMR chemical shifts according to eq 2. Solid lines in panel (b) show the concentration of all species according to the fitted equilibria (eqs 3 and 4). Error bars in (a) were estimated to ±0.05 ppm (see text). The error bars in (b) are calculated by propagation of error, based on an error in the DRS data of 2% of the static dielectric constant.

With these equilibrium concentrations, we advance our previous analysis^23^ and perform a combined analysis of both, the NMR chemical shifts and the DRS equilibrium concentrations to determine the association equilibria (SI eq S4). As can be seen in Figure 3, such combined fits describe both the NMR chemical shifts and the DRS detected concentrations well (fit parameters are listed in Table 1). More importantly, combining the results from both experiments improves fitting eq 2 as illustrated by the much narrower minima of the sum of squared deviations of the fit as shown in Figure S4 (see SI). As such, the combined approach reduces the parameter space when modeling the experimental data and thereby allows for a more accurate determination of the association equilibria.

### Effect of Solvent and Imine on Association Equilibria

**Figure 4:**
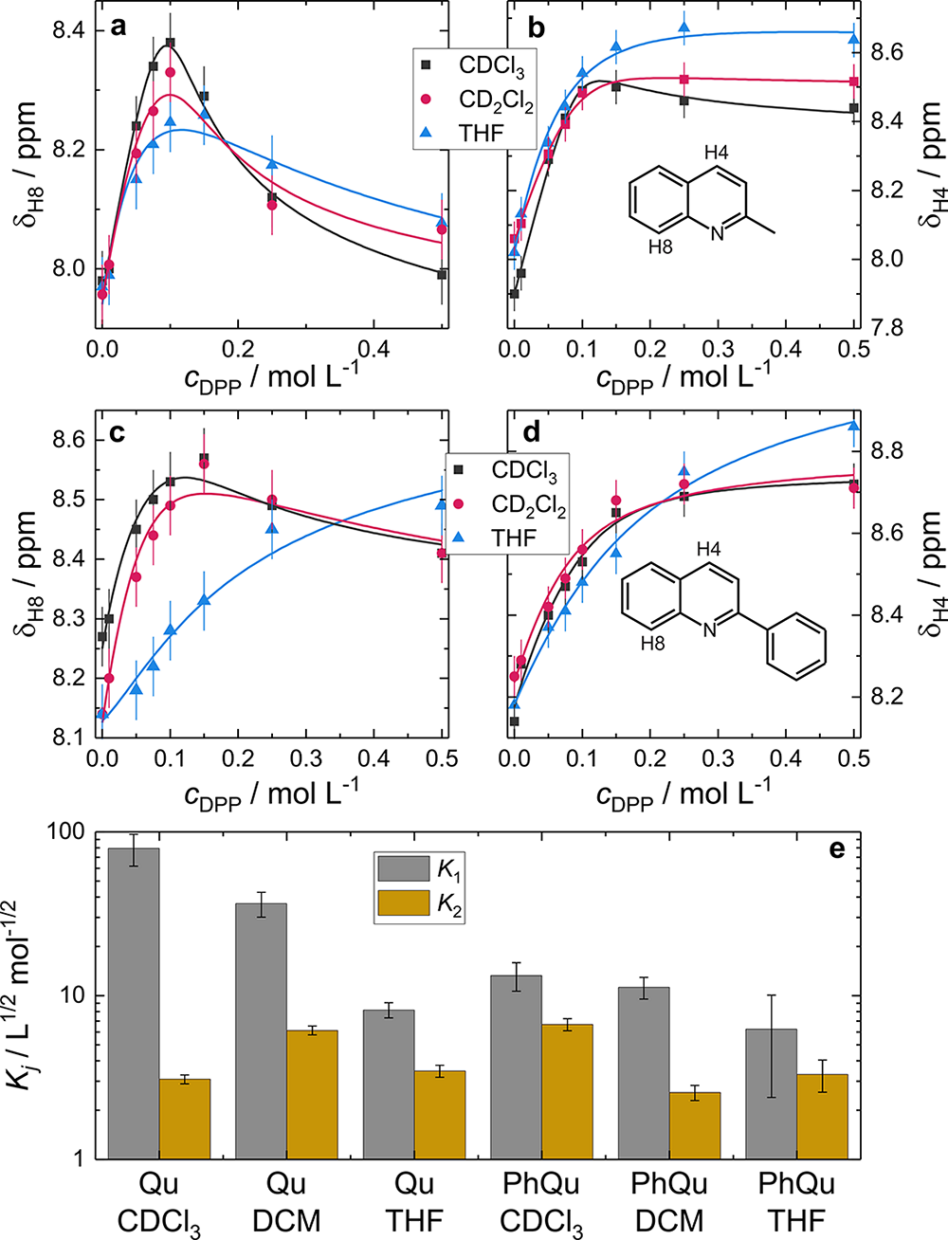
Chemical shifts of (a) H8 of Qu, (b) H4 of Qu, (c) H8 of PhQu, and (d) H4 of PhQu for solutions of Qu or PhQu (0.1 mol L^–1^) as a function of c_DPP_. Symbols show experimental data. Error bars were estimated to ±0.05 ppm (see text). Data for DCM in panels (a) and (b) are taken from ref (23). Solid lines show the combined fit of the NMR data and the DRS data (eqs 2–4 and S4). Insets of panels (b) and (d) display the molecular structures of Qu and PhQu, respectively. (e) Equilibrium constants, *K*_1_ and *K*_2_, as obtained from the combined NMR and DRS fit. The error bars in (e) correspond to a 10% increase of the sum of the squared deviations.

To study solvent effects, we investigate these equilibria for Qu and DPP in DCM, CDCl_3_, and THF (data for DCM are taken from ref (23).). The chemical shifts of H4 and H8 of Qu as a function of *c*_DPP_ in Figure 4a and b already reveal qualitative differences for the different solvents: The slope of the increase of δ for both protons at *c*_DPP_ < 0.1 mol L^–1^, where our data suggested that formation of ion-pairs dominates the observed changes, is highest for CDCl_3_, while for the solvent THF the increase in δ is less steep. Also at high *c*_DPP_, where according to our association model changes are due to multimer formation, the reduction of δ with increasing *c*_DPP_ is most pronounced for CDCl_3_, and weakest for THF.

To quantify the effect of the solvents on the association equilibria, we extract the values of *K*_1_ and *K*_2_ (see Figure 4e, Table 1), using the combined fit described above (solid lines in Figure 4, dielectric spectra and equilibrium concentrations are shown in SI Figures S5 and S6). In line with the qualitative trends of δ_H8_ in Figure 4a, we find the ion-pair formation constant (*K*_1_) to be highest in chloroform and slightly lower in DCM. In contrast to the chlorinated solvents, we find a significantly lower IP formation constant in THF (see Figure 4e). The values for *K*_2_ range from 3 to 7 L^1/2^ mol^–1/2^.

To explore the effect of the imine base on the solution equilibria, we performed the same set of experiments with 2-phenylquinoline (PhQu, see inset in Figure 4c), which can be hydrogenated with very high enantiomeric excess using asymmetric organo-catalysis.^58^ The chemical shift changes of H4 and H8 with *c*_DPP_ for PhQu (Figure 4c,d) in DCM and chloroform resemble those for Qu. The maxima of δ_H8_ are shifted to slightly higher *c*_DPP_ values for PhQu as compared to Qu. This shift of the maximum of the titration curve is indicative of different multimer formation equilibria relative to the ion-pair formation constants. In contrast to Qu, the chemical shift of H4 plateaus for all solvents at high *c*_DPP_. The decrease of the chemical shift of H8 at high *c*_DPP_ is less pronounced for PhQu as compared to Qu, which suggests that the formation of multimers affects the local chemical environment in the vicinity of PhQu’s N atom to a lesser extent. Conversely, the chemical shift of both H4 and H8 of PhQu in THF increases much less steeply as compared to DCM and CDCl_3_ and the NMR titration experiment exhibits a monotonic increase of both shifts (Figure 4c,d) with increasing acid concentration. In contrast to our findings for Qu, the chemical shifts do not fully plateau for PhQu, even for a 5-fold excess of DPP in THF. Thus, our results suggest that the association behavior of DPP and PhQu in THF differs from the other studied systems. On the basis of only the NMR data, for PhQu in THF only one association equilibrium could be inferred. Yet, the dielectric spectra exhibit the signatures of IP and M (see SI Figure S5): With increasing *c*_DPP_ the intensity of the low-frequency relaxation increases. Hence, our results also suggest that for PhQu in THF both ion-pairs and multimers with DPP are formed, yet, the concentrations of both species vary similarly with *c*_DPP_ (see SI Figure S6).

From the extracted association constants based on the combined DRS and NMR analysis (Figure 4e, Table 1), we find lower values of *K*_1_ for PhQu as compared to Qu for each studied solvent, respectively. Similar to Qu, we find the lowest *K*_1_ value for PhQu in THF, while *K*_1_ values are elevated in DCM and CDCl_3_. The *K*_2_ values are similar to those found for Qu, suggesting that in contrast to the IP formation, multimer formation is hardly affected by the base.

Overall, with the combined fit based on data from NMR and DRS experiments, we find that accounting for DPP homodimers results in a better description of the data from both experiments: Fits of the data including DPP homodimers (eqs 3 and 4), result in a markedly lower sum of the squared deviations of the fit from the data as compared to fits neglecting the acid dimerization (see SI Figure S7). For both bases we find consistently lower *K*_1_ values in THF as compared to DCM and chloroform. This solvent dependence cannot simply be explained by the dielectric permittivity of the studied solvents: On the basis of the solvents’ dielectric permittivities one would expect solvation of dipolar ion-pairs (and ionic species) to be most favorable in DCM and THF, which has the highest dielectric constant, ε, of all three solvents (DCM: ε = 8.9, THF: ε = 7.4, CHCl_3_: ε = 4.7).^59^ Hence, our results suggest that the pure “electrostatic” stabilization of the ion-pairs by the solvent is not the main factor determining solvent effects on the association equilibria. In turn, differences in specific interactions with the solvation may give rise to the observed trends: We find that the values of *K*_1_ are correlated with the hydrogen-bonding energy as determined by the Hansen solubility parameter, *δ*_h_:^60^ THF exhibits the highest value of *δ*_h_ = 3.9 cal^–1/2^ cm^3/2^, while DCM and CHCl_3_ exhibit lower *δ*_h_ values of 3.0 and 2.8 cal^–1/2^ cm^3/2^, respectively.^60^ As such, our results indicate hydrogen-bonding of the solvent has a more dramatic effect on the ion-pair formation equilibria.

The stronger IP formation constants of DPP with Qu as compared to PhQu is in line with the higher basicity of Qu relative to PhQu, as estimated from their aqueous pKb values.^61,62^ The similarity of the multimer formation constants for both bases may be somewhat unexpected. However, the similarity can be rationalized by the notion that multimer formation is primarily based on the interaction between the DPP^–^ anion of an IP with DPP, and is thus little affected by the more distant bases within a multimeric aggregate. The sensitivity of *K*_1_ to the nature of the base, together with the insensitivity of *K*_2_ makes both formation constants somewhat independent and they even become similar for PhQu in THF, which gives rise to the rather featureless titration curves in the NMR experiments (Figure 4c,d).

Relating our findings to catalytic performance of organo-phosphate Brønsted acid, our results are in line with our previous notion that the ion-pair formation (*K*_1_) is not decisive for enantioselectivity: In asymmetric catalysis the reported enantiomeric excess is similar in chloroform, DCM, and THF, despite the markedly reduced value of *K*_1_ in THF.^14^ Rather, as we have found previously,^14^ dissociation of ion-pairs into free ions diminishes enantioselectivity.^14^ Conversely, we find the trends in *K*_1_ to parallel the catalytic yields: the reported yields in organo-phosphoric acid catalyzed hydrogenations for PhQu are lower in THF as compared to DCM and CHCl_3_.^15^ This similarity suggests that the ion-pair formation constant plays an important role for the overall conversion. Given that the difference between the acidity of organo-phosphate Brønsted acid catalysts and the basicity of the imine influences *K*_1_ and also reaction rates,^8^ a high ion-pair formation constant likely accelerates reaction kinetics and thereby prevents incomplete or undesired chemical conversion in these hydrogenation reactions.

The similarity of the multimer formation constants for the herein studied systems makes a straightforward correlation to catalytic performance challenging, as at catalytic conditions little multimers are formed. Yet, the differences in the chemical shifts at high *c*_DPP_ (Figure 4) among the studied samples, indicates both, a solvent and base dependence of the electronic structure of the base within the multimers. These differences may result in different reaction pathways, which eventually can alter the enantiomeric excess as has been recently reported.^32^ Our results show that multimer formation is rather independent of the base, which suggests that multimers are in particular relevant in acid base mixtures, for which ion-pair formation is weak. In turn, solvents and bases with weak ion-pair formation should favor the multimeric reactive intermediates, which can be exploited in catalysis.^31,32^

## Conclusions

We present a combined approach to obtain association equilibria of acids and bases from NMR and DRS titration experiments. We use the NMR chemical shift of the base to detect the variation of the chemical environment of the base. To lift the ambiguity due to the correlation between the concentration of the aggregates and their associated chemical shift when modeling the data, we use DRS to determine the equilibrium concentrations of the aggregates at elevated acid concentrations. Using the combination of both experiments, improves the convergence when modeling the data and reduced the impact of systematic errors at low *c*_DPP_ for DRS and at high *c*_DPP_ for NMR. We find evidence for the formation of ion-pairs and multimers in all studied solutions containing DPP and imine bases. We show that for the association of DPP with Qu and PhQu, DPP dimerization has to be taken into account to accurately describe the observables from both experiments. Among the three studied solvents, we find the formation constants of ion-pairs from DPP and imine bases to be highest in CDCl_3_, slightly lower in DCM, and markedly lower in THF. The association of an additional DPP molecule to an ion-pair to form a multimer is similar in all studied solvents. Comparison of the two studied imines shows that the interaction of DPP with Qu is stronger than with PhQu, which is in line with the aqueous basicity of both imines. Comparison to reported catalytic efficiencies, where DPP-like acids are used to catalyze conversion of imine bases, indicates that the solvent’s effect on the ion-pair association strength correlates with the reaction yield. This correlation can be explained by enhanced reaction rates for strong ion-pair formation. Our results suggest that multimer formation equilibria are rather insensitive to the nature of the base and the solvent. As such, ion-pair formation and multimer formation are somewhat independent equilibria. Hence, these equilibria can potentially be tuned such that multimer formation is enhanced, which can pave the way to novel catalytic pathways.

## Supporting Information Available

Chemical shifts of H3, modeling of only the NMR data, detailed analysis of the dielectric relaxation experiments, and the details of the combined NMR and DRS fit (PDF)
