# Supplementary material for: Association Equilibria of Organo-Phosphoric Acids with Imines from a Combined Dielectric and Nuclear Magnetic Resonance Spectroscopy Approach
Source: Anal Chem. 2021 Feb 18;93(8):3914–21. doi: 10.1021/acs.analchem.0c04669 (PMC7931174; doi:10.1021/acs.analchem.0c04669)
Supplement: Supplementary file 1 — ac0c04669_si_001.pdf [file ac0c04669_si_001.pdf]

Supporting Information

Association equilibria of organo-phosphoric acids with imines from a combined dielectric and nuclear magnetic resonance spectroscopy approach

Christian Dreier, Leon Prädel, Amelie A. Ehrhard, Manfred Wagner, Johannes Hunger*

Max Planck Institute for Polymer Research, Department for Molecular Spectroscopy, Ackermannweg 10, 55128 Mainz, Germany

*email: hunger@mpip-mainz.mpg.de

Table of contents

Chemical shifts of the proton H3 S2

Modelling only the NMR chemical shifts S2

Analysis of the dielectric spectra S4

Combined DRS and NMR fit S5

Supporting References S9

Chemical shifts of the proton H3

As described in the main text, we use the chemical shift of the residual solvent protons for referencing the NMR spectra. These shifts of the solvents may however also be affected by the addition of DPP. In Figure S1 we show the chemical shift of H3 of Qu or PhQu as a function of c_{DPP}, which is – within experimental error – constant at $c_{DPP} \geq 0.1$ mol L^{-1}. This insensitivity of δ_{H3} to a large excess of DPP suggests that also the protons of the solvent are rather insensitive to the added DPP.

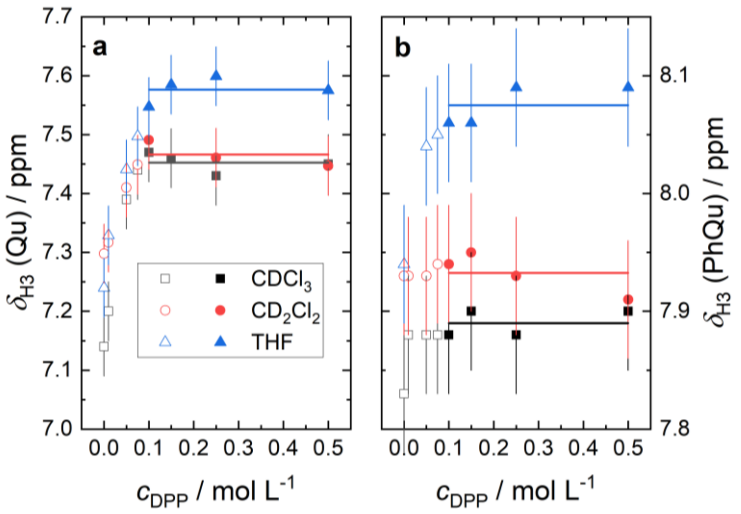

Figure S1: Chemical shift of H3 of 0.1 mol L^{-1} (a) Qu and (b) PhQu as a function of c_{DPP}. Open symbols show data at c_{DPP} < 0.1 mol L^{-1} and filled symbols depict data at $c_{DPP} \geq$ 0.1 mol L^{-1}. The solid lines show the average values at $c_{DPP} \geq 0.1$ mol L^{-1}. Error bars were estimated to ±0.05 ppm to account for systematic errors due to medium effects.

Modelling only the NMR chemical shifts

As described in the main text, we test modelling of the association equilibria based on K_1 and K_2 (eqs. 2-4 of the main manuscript), or based on K'_1 and K'_2 (see main text). This is achieved by optimizing the sum of the squared deviations, χ_{NMR}:

$$\chi_{\text{NMR}} = \frac{\sum\left(\left(\delta(\text{H8})_{\text{exp}} - \delta(\text{H8})_{\text{fit}}\right)/\Delta_{\text{NMR}}\right)^2}{N_{\text{NMR}}} + \frac{\sum\left(\left(\delta(\text{H4})_{\text{exp}} - \delta(\text{H4})_{\text{fit}}\right)/\Delta_{\text{NMR}}\right)^2}{N_{\text{NMR}}} \tag{S1}$$

where N_{NMR} is the number of data points. Δ_{NMR}, are the errors in the experimentally determined chemical shifts, δ_{exp}. δ_{fit} corresponds to the fit according to eq 2 of the main manuscript. χ_{NMR} is optimized using, δ_{Qu}, δ_{IP}, δ_M, K_1, and K_2 (or K'_1, and K'_2) as adjustable parameters. Yet, comparing the model taking DPP_2 dimerization into account (K_1 and K_2) to the model neglecting dimerization (K'_1 and K'_2) provides no evidence for clearly lower χ_{NMR} values for one of the two models (Figure S2).

The insensitivity of the fit quality to the association model can be explained by correlation of the adjustable parameters. As described in the main manuscript, variation of the association

constant K_1 can be – predominantly – compensated by a variation of the chemical shift of the ion pair, δ_{IP}, when fitting eq 2 (see main manuscript) to the experimentally determined chemical shifts of Qu's protons. This is illustrated in Figure S3, where we show the variation of δ_{IP} when fitting eq 4 to the data for Qu in $CDCl_3$ for different values of K_1.

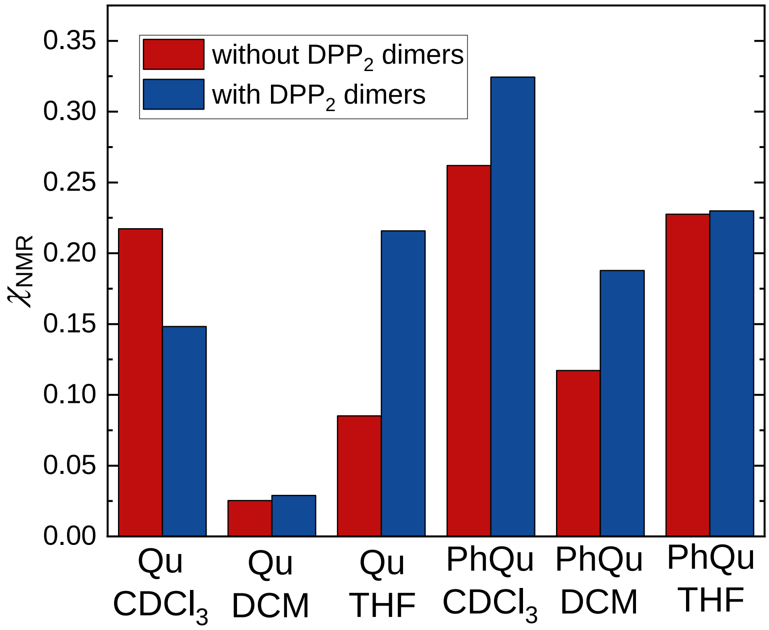

Figure S2: Comparison of the fit quality (χ_{NMR}) when fitting eq 2 to the NMR chemical shifts. Blue columns correspond to fits taking DPP dimerization into account (eqs 3-4), while red columns show fits based on free DPP monomers in solution.

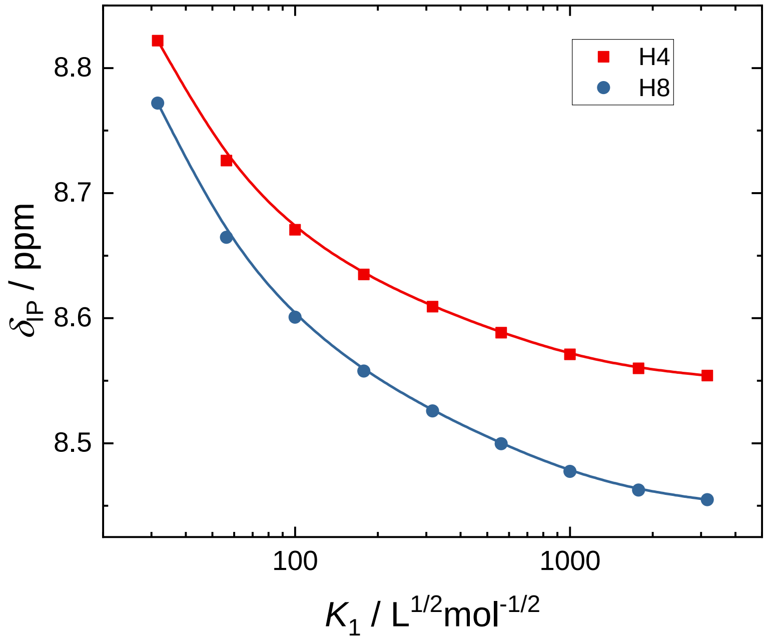

Figure S3. Chemical shift of the ion-pairs, δ_{IP}, as obtained from fitting eq 2 (main manuscript) to the data shown in Figure 1 of the main manuscript as a function of constrained values of K_1. Selected fits are shown as solid lines in Figure 1 of the main manuscript.

Analysis of the dielectric spectra

In order to quantify the concentration of ion-pairs and multimers in solution, we analyse the dielectric spectra of the imine – DPP mixtures. Therefore, we fit a relaxation model to the experimental spectra. In line with our previous study, we find that a combination of three Debye type equations[1] can describe the experimental spectra (see also Figure 2 of the main manuscript). In this model the relaxation of the multimers (M), ion-pairs (IPs), and the solvent (solv) is each modelled with a separate Debye relaxation. Each relaxation is characterized by its relaxation strength (S_j) and relaxation time (τ_j). Using this model, we assume uncorrelated dipolar relaxation of the different molecules and/or molecular aggregates. All polarizations at frequencies (ν) higher than the experimentally covered frequency range are modelled by the limiting permittivity at infinite frequencies, ε_∞. Ohmic losses due to translational polarization are taken into account by the last term of eq S1, with the d.c. conductivity κ assumed to be real and independent of frequency. ε_0 is the vacuum permittivity:

$$\hat{\varepsilon}(\nu) = \frac{S_\mathrm{M}}{1 + 2\pi\mathrm{i}\nu\tau_\mathrm{M}} + \frac{S_\mathrm{IP}}{1 + 2\pi\mathrm{i}\nu\tau_\mathrm{IP}} + \frac{S_\mathrm{solv}}{1 + 2\pi\mathrm{i}\nu\tau_\mathrm{solv}} + \varepsilon_\infty + \frac{\kappa}{2\pi\mathrm{i}\nu\varepsilon_0} \quad \text{(S2)}$$

Based on the Cavell equation

$$\frac{2\varepsilon_\mathrm{s} + 1}{\varepsilon_\mathrm{s}} \cdot S_j = \frac{N_\mathrm{A}[j]}{k_\mathrm{B} T \varepsilon_0} \cdot {\mu_j}^2 \quad \text{(S3)}$$

the obtained relaxation amplitudes, S_j are directly related to concentration of the species, $[j]$, in solution[1–3] and their (squared) electrical dipole moment, μ_j. In eq S3, $\varepsilon_\mathrm{s}(= S_\mathrm{M} + S_\mathrm{IP} + S_\mathrm{solv} + \varepsilon_\infty)$ is the static dielectric constant, N_A Avogadro's constant, k_B the Boltzmann constant, and T the thermodynamic temperature.

To relate the observed relaxation strengths to the concentration of the relaxing species, their electrical dipole moment is required. Here, we assume the dipole moment of the ion-pair and of the multimer to be the same $\mu_\mathrm{IP,M} = \mu_\mathrm{IP} = \mu_\mathrm{M}$. This assumption is supported by *ab initio* calculations and experiments for DPP-Qu aggregates in DCM, which both indicate very similar dipole moments for both species.[1] Further *ab initio* calculations (DFT[4–6] / B3LYP 6-31+G(d)[7–9] as implemented in Gaussian 09[10]) for Qu-DPP ion-pairs and Qu-(DPP)$_2$ trimers in continuum solvents (PCM) suggest very similar dipole moments in the other studied solvents: $CHCl_3$ (μ_IP = 14.4 D, μ_M = 13.1 D), THF (μ_IP = 15.0 D, μ_M = 15.0 D).

Given that all imine bases form either ion-pairs or multimers ($c_\mathrm{Qu} \approx [\mathrm{IP}] + [\mathrm{M}]$), which can be justified for an excess of DPP, $\mu_\mathrm{IP,M}$ is obtained from the analytical concentration of base and from the combined relaxation strength $S_\mathrm{IP,M} = S_\mathrm{IP} + S_\mathrm{M}$ using eq S3. Here, we use the spectra with a five-fold excess of DPP to determine the dipole (see Table S1). These values allow obtaining the equilibrium concentrations [IP] and [M] from the experimental S_IP and S_M values (eq S3), respectively, at all three studied concentrations of each measurement series.

Table S1. Calculated combined dipole moments for ion-pairs and multimers.

Solvent	$\mu_{IP,M}$(Qu) [D]	$\mu_{IP,M}$(PhQu) [D]
DCM	23.6	25.2
$CHCl_3$	19.2	23.3
THF	20.8	23.9

Combined DRS and NMR fit

As described in the main manuscript, we perform a combined fit of the experimental chemical shifts for H8 ($\delta(\mathrm{H8})_{\mathrm{exp}}$) and H4 ($\delta(\mathrm{H4})_{\mathrm{exp}}$) and the experimental concentrations of ion-pairs $[\mathrm{IP}]_{\mathrm{DRS}}$ and multimers $[\mathrm{M}]_{\mathrm{DRS}}$ based on the association equilibria as outlined in the main manuscript. To model the chemical shift of both protons we use eq 2 (main manuscript). In the combined fit, we optimize the weighted sum of the square deviations from both experiments:

$$\chi_{\mathrm{DRS+NMR}} = \frac{\sum\left(\left(\delta(\mathrm{H8})_{\mathrm{exp}} - \delta(\mathrm{H8})_{\mathrm{fit}}\right)/\Delta_{\mathrm{NMR}}\right)^2}{N_{\mathrm{NMR}}} + \frac{\sum\left(\left(\delta(\mathrm{H4})_{\mathrm{exp}} - \delta(\mathrm{H4})_{\mathrm{fit}}\right)/\Delta_{\mathrm{NMR}}\right)^2}{N_{\mathrm{NMR}}} + \frac{\sum\left(\left([\mathrm{M}]_{\mathrm{DRS}} - [\mathrm{M}]\right)/\Delta_{\mathrm{M,DRS}}\right)^2}{N_{\mathrm{DRS}}} + \frac{\sum\left(\left([\mathrm{IP}]_{\mathrm{DRS}} - [\mathrm{IP}]\right)/\Delta_{\mathrm{IP,DRS}}\right)^2}{N_{\mathrm{DRS}}} \tag{S4}$$

where N_{DRS} is the number of data points of DRS experiments. $\Delta_{\mathrm{M,DRS}}$, and $\Delta_{\mathrm{IP,DRS}}$ are the errors in $[\mathrm{M}]_{\mathrm{DRS}}$, and $[\mathrm{IP}]_{\mathrm{DRS}}$, respectively. $\chi_{\mathrm{DRS+NMR}}$ is optimized using δ_{Qu}, δ_{IP}, δ_M, K_1, and K_2 (or K'_1, and K'_2) as adjustable parameters. As can be seen from Figure S4, using the data from both experiments provides much steeper minima in $\chi_{\mathrm{DRS+NMR}}$, as compared to the fit based on only NMR data (χ_{NMR}).

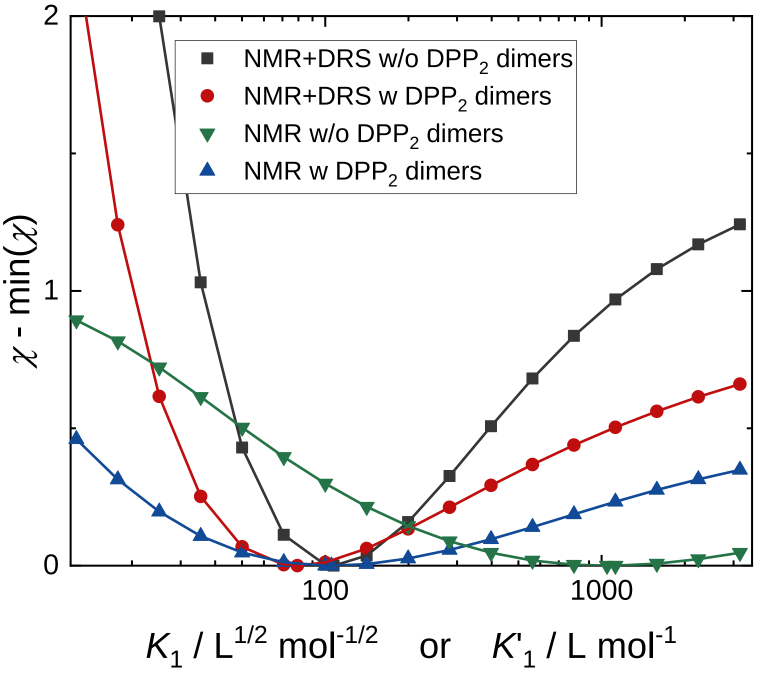

Figure S4: Comparison of the relative fit quality for DPP + Qu in chloroform as a function of contained ion-pair formation constants (K_1 or K'_1) when modelling only NMR data (χ_{NMR}) or boths NMR and DRS data ($\chi_{DRS+NMR}$).

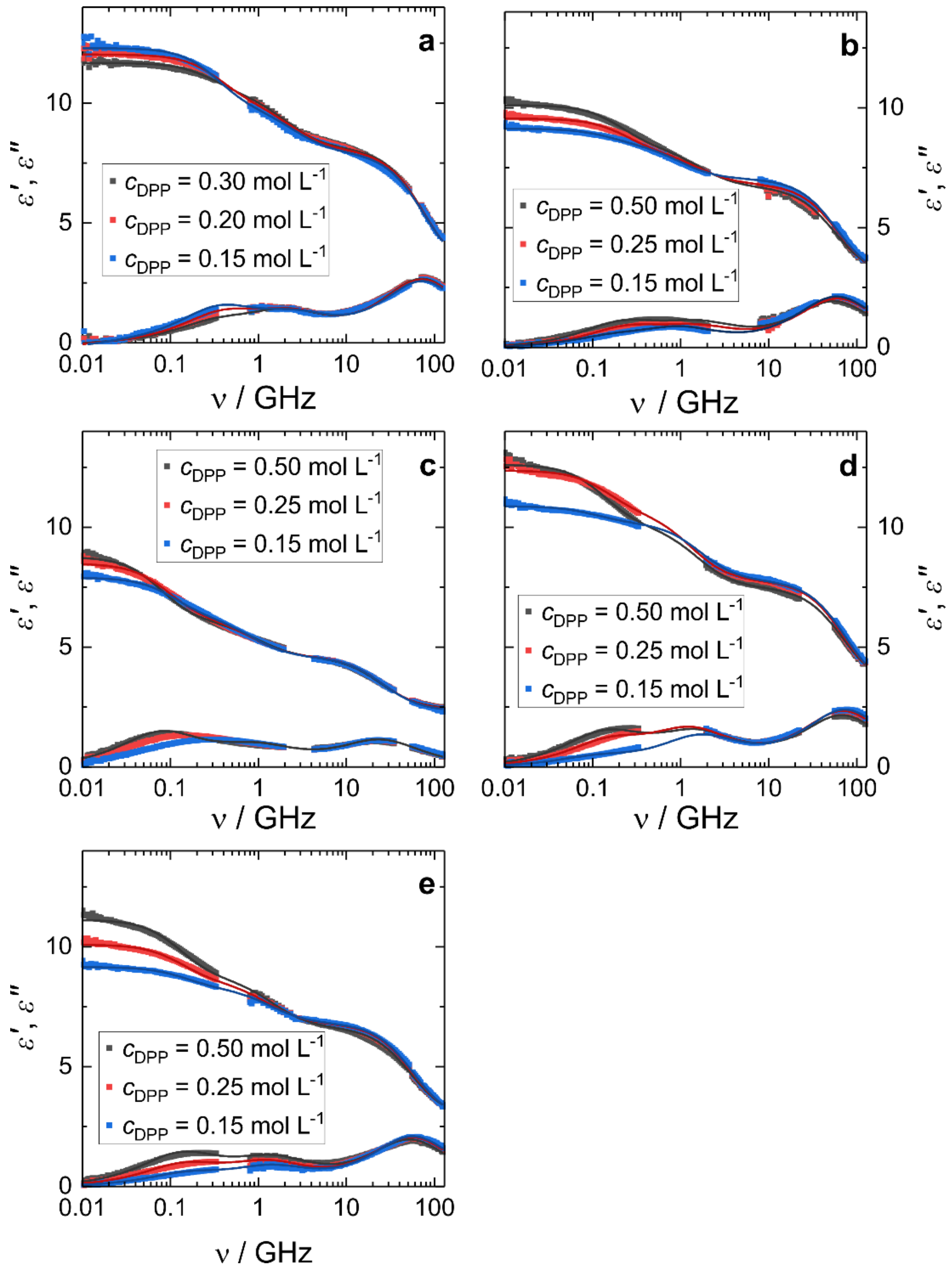

Figure S5. DRS spectra for (a) Qu in DCM, (b) Qu in THF, (c) PhQu in $CHCl_3$, (d) PhQu in DCM, and (e) PhQu in THF. The imine concentration is kept constant at 0.1 mol L^{-1} in all samples. The data shown in panel a) are taken from ref. 1. Symbols correspond to experimental spectra, solid lines show fits of eq S2 to the data.

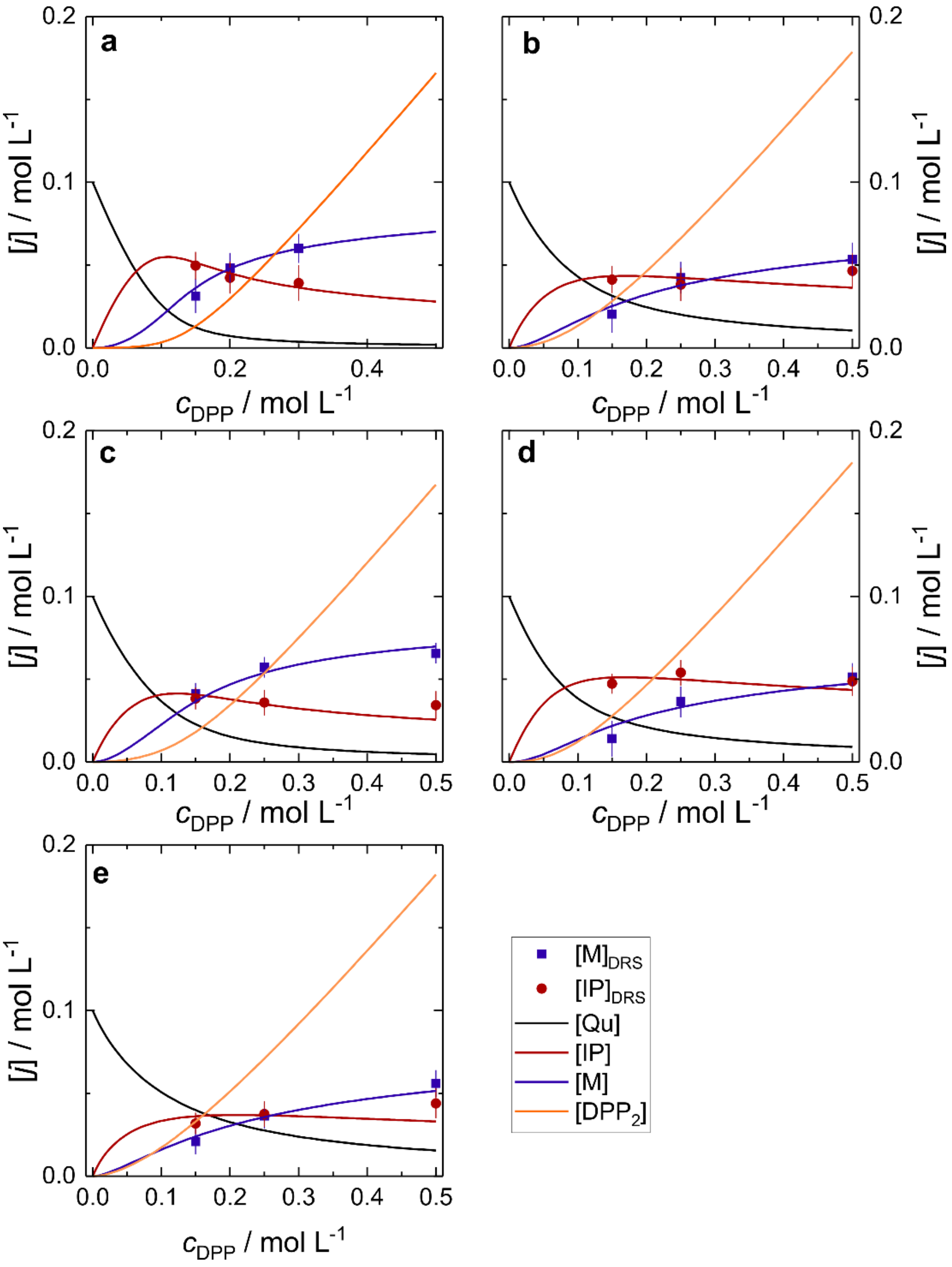

Figure S6. Concentration of multimers and ion-pairs (symbols) as extracted from the DRS experiments together with the concentration of all species (solid lines) according to the fitted equilibria (eqs 3 & 4, main manuscript) for (a) Qu in DCM, (b) Qu in THF, (c) PhQu in $CHCl_3$, (d) PhQu in DCM, and (e) PhQu in THF. Error bars are calculated assuming the error of the relaxation strengths to 2 % of ε_s.

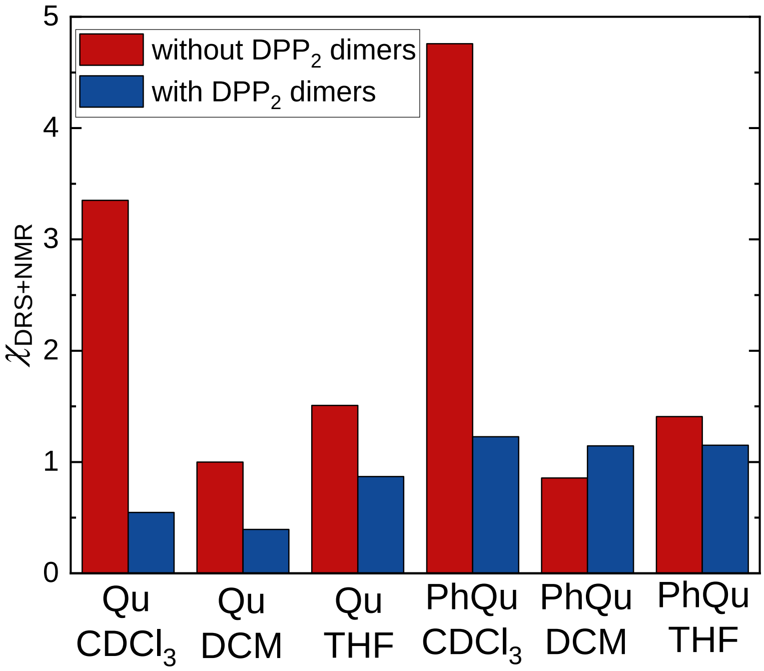

Figure S7. Comparison of the fit quality ($\chi_{NMR+DRS}$) for simultaneously fitting the NMR and DRS data. Blue columns correspond to fits taking DPP dimerization into account (eqs 3-4), while red columns show fits based on free DPP monomers in solution.

Supporting References

(1) Malm, C.; Kim, H.; Wagner, M.; Hunger, J. Complexity in Acid-Base Titrations: Multimer Formation Between Phosphoric Acids and Imines. *Chem. - A Eur. J.* **2017**, *23* (45), 10853–10860. https://doi.org/10.1002/chem.201701576.

(2) Kim, H.; Sugiono, E.; Nagata, Y.; Wagner, M.; Bonn, M.; Rueping, M.; Hunger, J. Role of Ion-Pairs in Brønsted Acid Catalysis Supporting Information. *ACS Catal.* **2015**, 6630–6633. https://doi.org/10.1021/acscatal.5b01694.

(3) Cavell, E. A. S.; Knight, P. C.; Sheikh, M. A.; A., S. M.; Sheikh, M. A. Dielectric Relaxation in Non Aqueous Solutions. Part 2. Solutions of Tri(n-Butyl)Ammonium Picrate and Iodide in Polar Solvents. *Trans. Faraday Soc.* **1971**, *67*, 2225–2233. https://doi.org/10.1039/TF9716702225.

(4) Parr, R. G.; Yang, W. Density-Functional Theory of the Electronic Structure of Molecules. *Annu. Rev. Phys. Chem.* **1995**, *46* (1), 701–728. https://doi.org/10.1146/annurev.pc.46.100195.003413.

(5) Hohenberg, P.; Kohn, W. Inhomogeneous Electron Gas. *Phys. Rev.* **1964**, *136* (3B), B864–B871. https://doi.org/10.1103/PhysRev.136.B864.

(6) Kohn, W.; Sham, L. J. Self-Consistent Equations Including Exchange and Correlation Effects. *Phys. Rev.* **1965**, *140* (4A), A1133–A1138. https://doi.org/10.1103/PhysRev.140.A1133.

(7) Becke, A. D. Density-Functional Thermochemistry. III. The Role of Exact Exchange. *J. Chem. Phys.* **1993**, *98* (7), 5648. https://doi.org/10.1063/1.464913.

(8) Petersson, G. A.; Bennett, A.; Tensfeldt, T. G.; Al-Laham, M. A.; Shirley, W. A.; Mantzaris, J. A Complete Basis Set Model Chemistry. I. The Total Energies of Closed-shell Atoms and Hydrides of the First-row Elements. *J. Chem. Phys.* **1988**, *89* (4), 2193–2218. https://doi.org/10.1063/1.455064.

(9) Petersson, G. A.; Al-Laham, M. A. A Complete Basis Set Model Chemistry. II. Open-shell Systems and the Total Energies of the First-row Atoms. *J. Chem. Phys.* **1991**, *94* (9), 6081–6090. https://doi.org/10.1063/1.460447.

(10) Frisch, M. J.; Trucks, G. W.; Schlegel, H. B.; Scuseria, G. E.; Robb, M. A.; Cheeseman, J. R.; Scalmani, G.; Barone, V.; Mennucci, B.; Petersson, G. A.; Nakatsuji, H.; Caricato, M.; Li, X.; Hratchian, H. P.; Izmaylov, A. F.; Bloino, J.; Zheng, G.; Sonnenberg, J. L.; Hada, M.; Ehara, M.; Toyota, K.; Fukuda, R.; Hasegawa, J.; Ishida, M.; Nakajima, T.; Honda, Y.; Kitao, O.; Nakai, H.; Vreven, T.; Montgomery, J. A. J. A.; Peralta, J. E.; Ogliaro, F.; Bearpark, M.; Heyd, J. J.; Brothers, E.; Kudin, K. N.; Staroverov, V. N.; Keith, T.; Kobayashi, R.; Normand, J.; Raghavachari, K.; Rendell, A.; Burant, J. C.; Iyengar, S. S.; Tomasi, J.; Cossi, M.; Rega, N.; Millam, J. M.; Klene, M.; Knox, J. E.; Cross, J. B.; Bakken, V.; Adamo, C.; Jaramillo, J.; Gomperts, R.; Stratmann, R. E.; Yazyev, O.; Austin, A. J.; Cammi, R.; Pomelli, C.; Ochterski, J. W.; Martin, R. L.; Morokuma, K.; Zakrzewski, V. G.; Voth, G. A.; Salvador, P.; Dannenberg, J. J.; Dapprich, S.; Daniels, A. D.; Farkas, O.; Foresman, J. B.; Ortiz, J. V.; Cioslowski, J.; Fox, D. J.; Farkas; Foresman, J. B.; Ortiz, J. V.; Cioslowski, J.; Fox, D. J. *Gaussian 09, Revision B.01*; Wallingford CT.
